# Supplementary material for: A three-dimensional view of structural changes caused by deactivation of fluid catalytic cracking catalysts
Source: Nat Commun. 2017 Oct 9;8:809. doi: 10.1038/s41467-017-00789-w (PMC5634498; doi:10.1038/s41467-017-00789-w)
Supplement: Supplementary file 1 — Supplementary Information [file 41467_2017_789_MOESM1_ESM.pdf]

Supplementary Figures

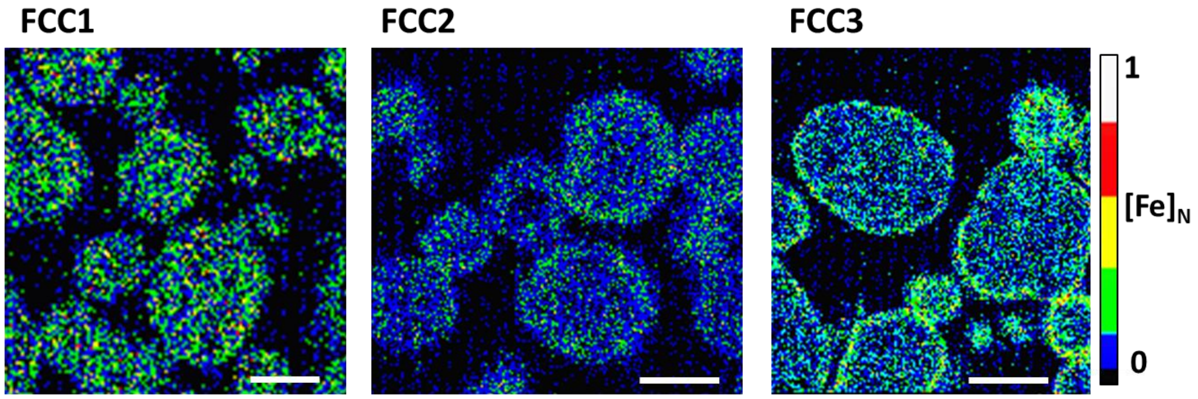

Supplementary Figure 1: Localization of Iron within FCC Particles. FCC particles were embedded in epoxy resin, surface polished and subjected to electron probe microanalysis (EPMA) to visualize the distribution and relative abundance of iron, spatial resolution ~ 1 µm. From the EPMA micrographs we can clearly see a shift from the homogenous distribution of iron in the pristine sample (FCC1), mainly originating from the naturally iron rich clay, to the accumulation of feedstock introduced iron impurities in the outer-shell of the particle. Presented colour map is representative of the relative iron concentration in the field of view during image acquisition. Scale bars are 50 µm.

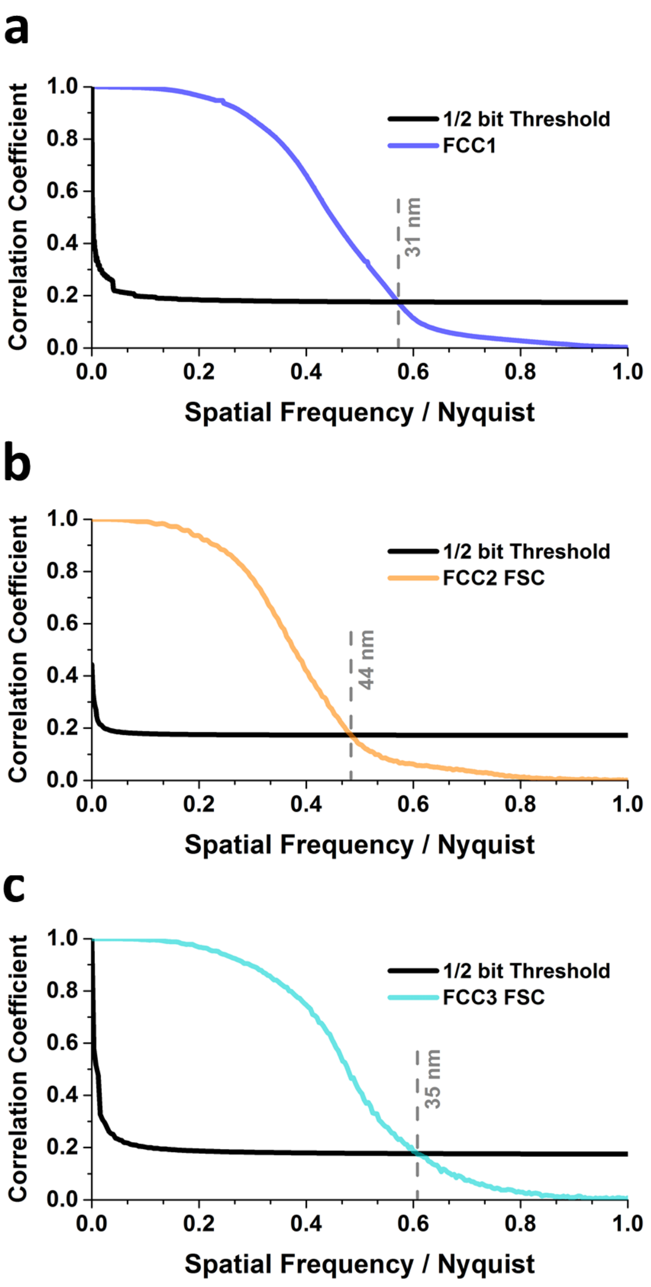

Supplementary Figure 2: Spatial Resolution of Ptychographic Tomograms. Shown are Fourier shell correlations (FSC) line outs, computed using the full dataset of projections of FCC1 (a) FCC2 (b) and FCC3 (c). The selected threshold criteria for the FSC is the ½ bit criterion.[1] Voxel sizes are $(18.2\ nm)^3$ (FCC1), $(21.3\ nm)^3$ (FCC2) and $(21.4\ nm)^3$ (FCC3). The spatial resolution estimate of the FCC1 tomogram is 31 nm. The spatial resolution of FCC2 is estimated to be 44 nm. The spatial resolution of FCC3 is estimated to be 35 nm.

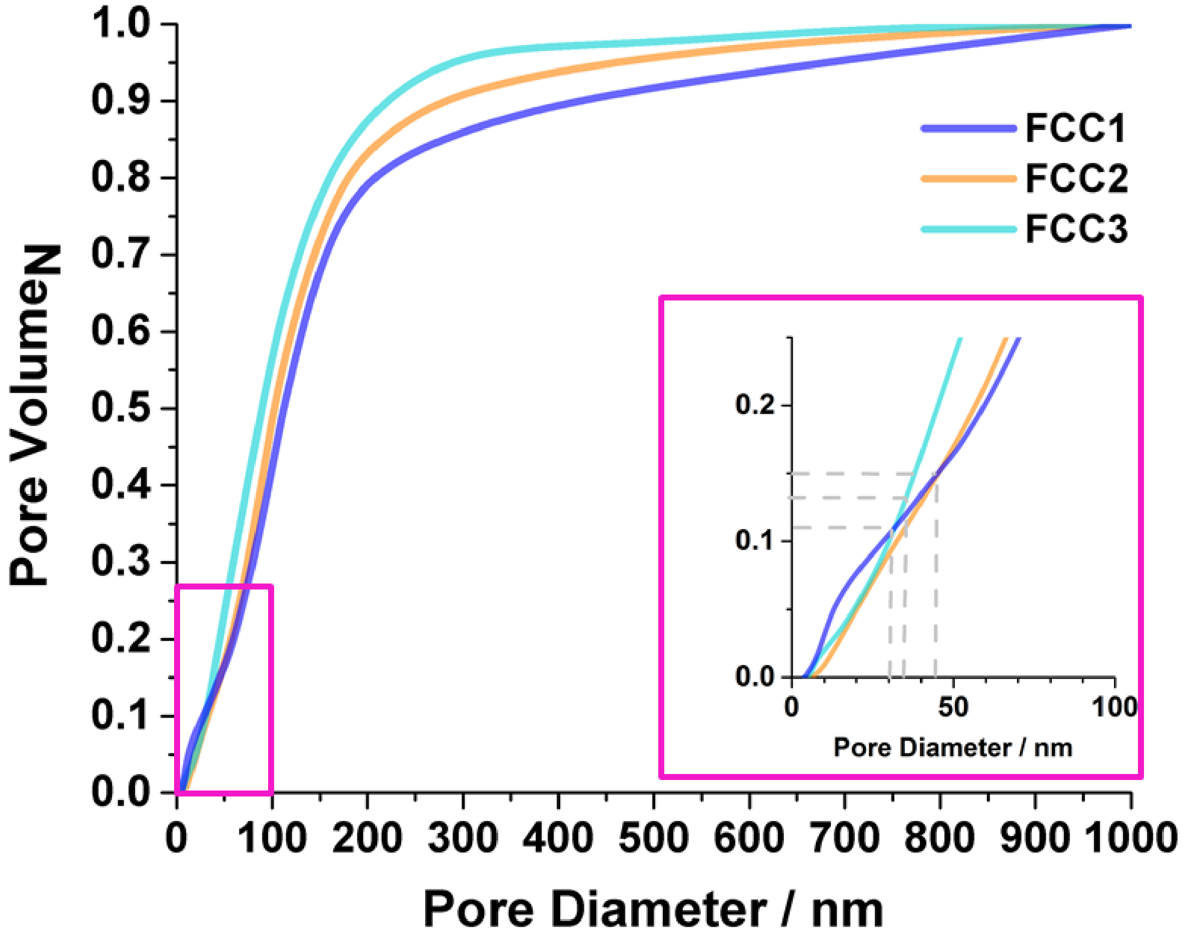

Supplementary Figure 3: Mercury Intrusion Porosimetry. Shown are the mercury intrusion porosimetry determined pore volume distributions of FCC1, FCC2 and FCC3. The grey dotted lines indicate the estimated spatial resolution of ptychographic tomograms. Considering that intrusion measurements by nature are defined by pore throats,[2] ascribing selected pore network volumes that lie behind a pore throat to the pore throat itself i.e. overestimating the volume of smaller pores we referred to literature[3] reported BET derived pore volume distribution of pores 2-100 nm in diameter, compromising 30% of the total MIP determined pore volume, to obtain a second evaluation of the volume distribution of smaller pores. The BET determined pore volume distribution presented by Wallenstein et al.[3] suggest that on average only ~30 vol.% of BET measured pores have a diameter smaller than the estimated spatial resolution. A comparison of PXCT resolution estimates with the apparent pore size distribution (BET+MIP) then reveals that PXCT tomograms probe on average ~90% of the total pore volume.

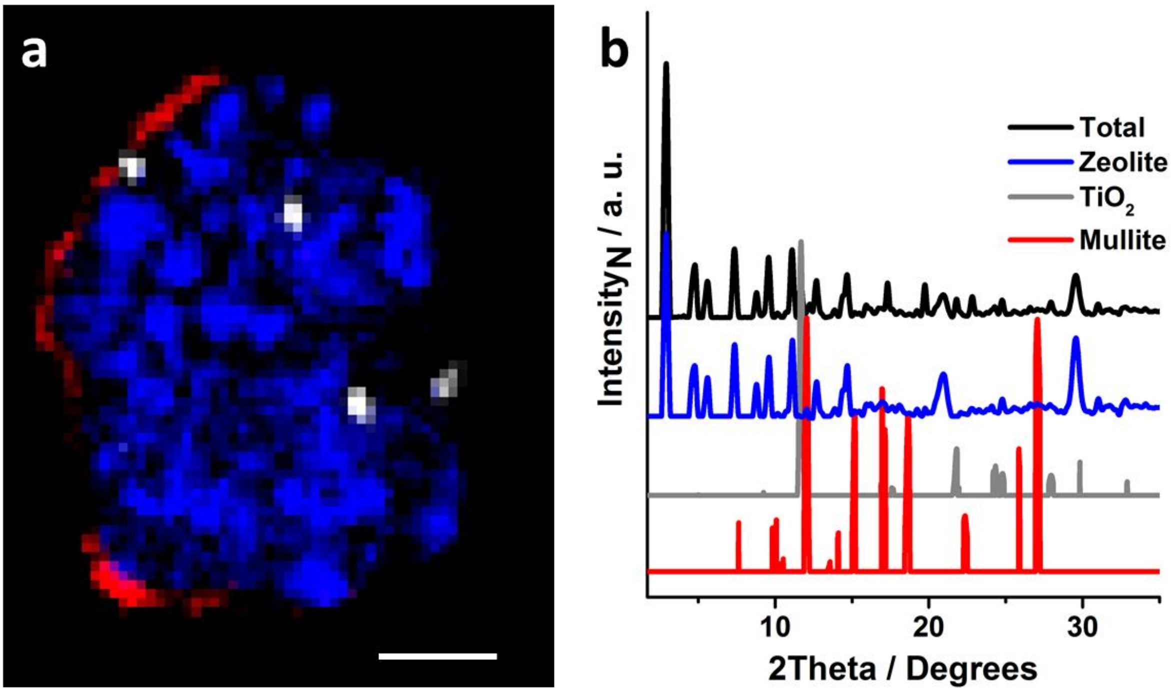

Supplementary Figure 4: Distribution of Crystalline Components and Azimuthal Integrated Diffraction Patterns. Shown in (a) is the detectable distribution of crystalline components within the FCC3 particle shown in Figure 6. These components are titania (anatase), zeolite (H-USY) and mullite ($Al_6Si_2O_{13}$). Scale bar is 20 µm. Voxel size is (1.5 µm)^3. The corresponding diffraction patterns are shown in (b). X-rays of 17.3 keV were used. Mullite is a thermal phase transformation product of metakaolin,[4] the matrix component. Its selective detection within the iron enriched shell provides further support to Hunt's proposition of surface melting and vitrification cycles.[5] The extremely low diffraction intensity of the identified mullite is noted.

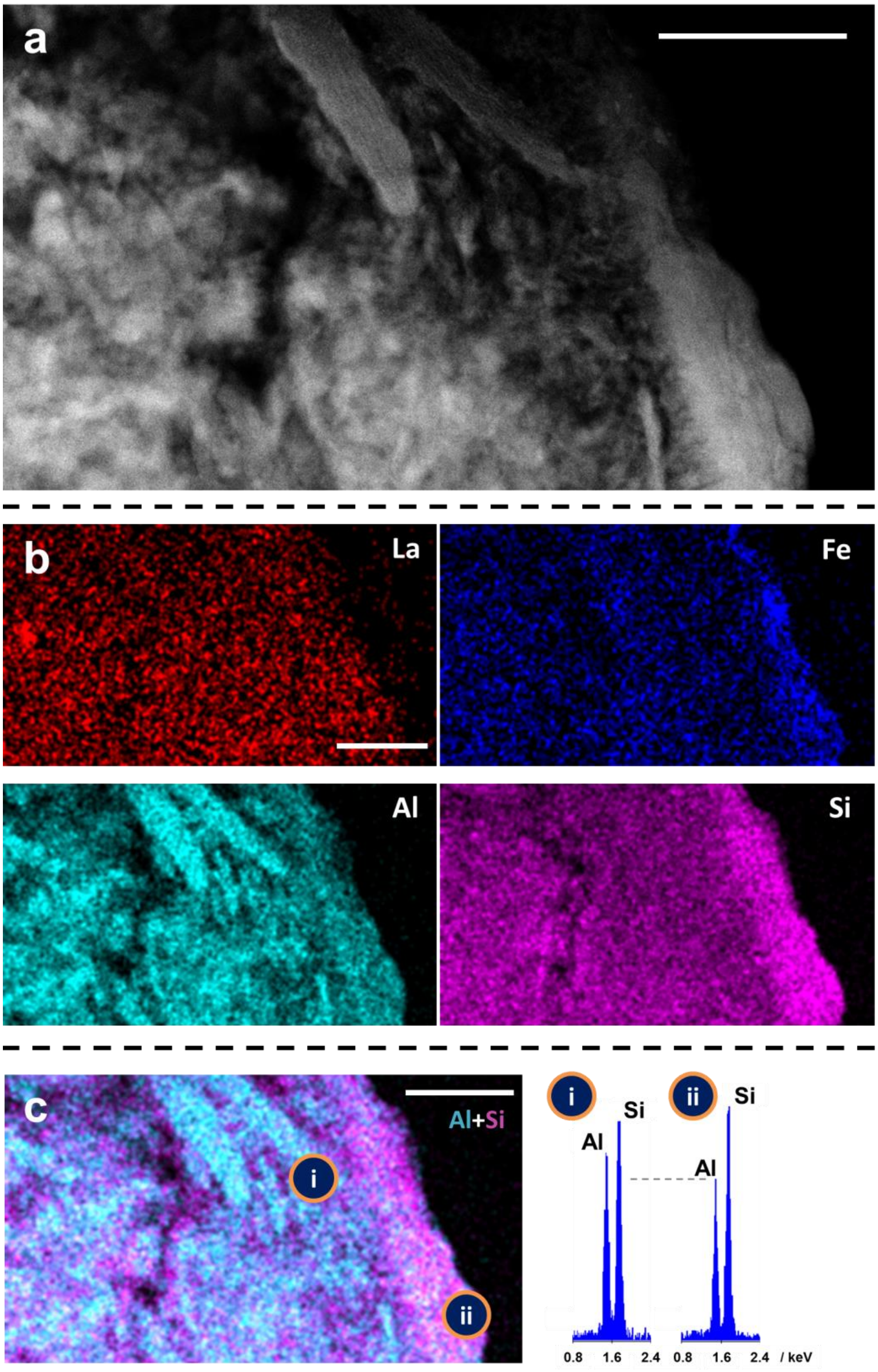

Supplementary Figure 5: FCC3 Electron Microscopy Analysis. Shown is a combination of electron micrograph and energy-dispersive X-ray (EDX) spectroscopy maps collected across a thin section of an FCC3 particle. Shown in (a) is a scanning transmission electron micrograph recorded with a high-angle annular dark field detector of a thin cut section. Presented in (b) are EDX spectroscopy maps of La, Fe, Al and Si. (c) Overlay of Al and Si EDX maps. Further provided in (c) are selected sections of the energy dispersive X-ray spectra of clay located within the particle (i) and ASA in the newly formed shell (ii). Observed increase in the Si/Al ratio and presence of lanthanum at the edge of the particle provides further support to the amorphization of zeolites close to the particle exterior. Scale bars are 2 µm.

Supplementary Table

Supplementary Table 1: Composition and Physical Properties of FCC Particles.

Sample	FCC1	FCC2	FCC3
**Bulk Analysis**			
**Composition/ wt.%**			
Al_2O_3	51.2	48.4	48.3
La_2O_3	2.06	1.90	1.80
TiO_2	0.95	1.01	1.22
Fe_2O_3	0.53*	0.72	1.60
Na_2O	0.24	0.34	0.42
Ni	0	0.23	0.13
V	0	0.51	0.11
**Unit Cell/ nm**			
Zeolite	2.451	2.429	2.430
**Surface Area/ m^2g^{-1}**			
Total	264	135	118
Total Loss%	-	49	55
Meso + Macropores	107 (41%)	53 (39%)	68 (58%)
Zeolite	157 (59%)	82 (61%)	50 (42%)
Zeolite Loss%	-	48	68
**Catalytic Activity/ wt.%**			
Conversion	88	64	56
Activity Loss %	-	27	36
**Ptychographic X-ray Computed Tomography**			
**Dimensions of the analysed part of the sample**			
Volume/ μm^3	3620	2480	4703
**Composition**	vol.%	vol./%	vol./%
Pores	22	23	24
Zeolite	53	55	52
Clay/ASA (Al_2O_2:SiO_2)	25	22	24
**Surface Area/ m^2g^{-1}**			
Total	132	79	93
Meso + Macropores	131	78	92
**▼Interfacial Area between Zeolite and Reaction Environment (Pores + Exterior)/ %total**			
Zeolite/ Environment	70	62	62
Zeolite/ Exterior	10	14	~2
Zeolite/ Pores	50	48	60

Bulk Analysis: Data provided by W. R. Grace. Sample composition was determined by inductively coupled plasma emission spectrometry (ICP). *~2 wt.% are natural to kaolin clay.[6] Catalytic activity was determined using an Advanced Cracking Evaluation (ACE) unit. **Ptychographic X-ray computed tomography:** Sample dimensions provided are based on the tomograms shown in Figure 2. ▼The interfacial area between zeolite and reaction environment represents the surface area of zeolite elements which could be potentially active. Disregarding the surface areas associated with micropores and assuming total pore assessability independent of composite isolating elements. Provided estimates are subject to finite image resolution and segmentation limitations, Figure 3a.

Supplementary Methods

Ptychographic X-ray Computed Tomography

Ptychographic X-ray computed tomography[7] is a lensless imaging technique in which the phase problem is solved by means of iterative phase retrieval algorithms. By solving the phase problem at different projection angles, ptychographic tomography retrieves the complex-valued transmissivity of the specimen providing tomograms of both phase and amplitude contrast whose resolution is limited by the angular spread of the specimen scattered intensity.[8, 9] Knowing both phase shifts and the chemical composition of the specimen it is possible to perform quantitative 3D density mapping.[10, 11]

Experimental Setup and Data Acquisition: Ptychographic X-ray computed tomography experiments were carried out at the cSAXS beamline of the SLS. The photon energy was either set to 7.3 keV (FCC1) or 6.2 keV (FCC2 and FCC3) using a double-crystal Si(111) monochromator. The horizontal aperture of slits located 22 m upstream of the sample was set to 20 μm in width. This was done to create a horizontal virtual source point coherently illuminating a Fresnel zone plate, the latter being either 150 μm (FCC1) or 170 μm (FCC2 and FCC3) in diameter with an outermost zone width of 60 nm.[12]

FCC1 was placed 1.2 mm downstream of the zone plate's focal point, resulting in an illumination probe of ~3.5 μm in diameter at the sample plane. **FCC2** was placed 1.2 mm downstream of the zone plate's focal point, resulting in an illumination probe of ~4 μm in diameter at the sample plane. **Coked FCC3** was placed 0.93 mm downstream of the zone plate's focal point. This results in an illumination probe of ~3.1 μm at the sample plane. Coherent diffraction patterns were acquired using a PILATUS 2M detector[13] with a 172 μm pixel size, 7.31 m (FCC1), 7.33 m (FCC2) or 7.34 m (FCC3) downstream of the sample. A He-flushed or an evacuated flight tube for FCC1 and FCC3 or FCC2 respectively, was positioned between sample and detector to reduce air scattering and absorption. Measurements were carried out using the positioning instrumentation described in Holler *et al.*[14, 15] FCC1 and the coked FCC3 were imaged at room temperature in air. FCC2 was imaged in an in-vacuum version of this setup at a temperature of -180°C in vacuum. Sampling positions were set using a Fermat spiral scanning grid[16] with an average step size of 1.2 μm (FCC1 and FCC2) and 1.3 μm (FCC3).

Tomography projections were acquired using a binary acquisition strategy as described by Kaestner *et al.*.[17] For **FCC1**, 1400 projections, of equal angular spacing were acquired across an angular range of 180º. Each projection was obtained by a ptychographic scan of 267 diffraction patterns, each with an exposure time of 0.1 second. The field of view covered in each projection was of 38 × 10 μm^2 (H × V). **FCC2**, 1000 projections, of equal angular spacing, were acquired across an angular range of 180º. Each projection was obtained by a ptychographic scan of 415 diffraction patterns, each with an exposure time of 0.1 second. The field of view covered in each projection was of 40 × 15 μm^2 (horizontal × vertical). **FCC3**, 2000 projections, of equal angular spacing, were acquired across an angular range of 180 º. Each projection was obtained by a ptychographic scan of 253 diffraction patterns, each with an exposure time of 0.1 second. The field of view covered in each projection was of 43 × 10 μm^2 (horizontal × vertical).

To compensate for the loss of information between individual detector modules we moved the detector perpendicular to the X-ray propagation direction by a distance slightly larger than the vertical and horizontal module gap widths after two successive projections.

The X-ray dose imparted to the specimens was on the order of 2×10^8 Gy.[18]

Image Reconstructions: From each diffraction pattern, a region of 400 × 400 pixels was used in the reconstructions. The resulting pixel sizes are $(18.2\ nm)^2$ (FCC1), $(21.3\ nm)^2$ (FCC2) and $(21.4\ nm)^2$ (FCC3) in the reconstructed projections. **FCC1**, reconstructions were obtained with 800 iterations of the difference map algorithm[9] followed by 200 iterations of maximum likelihood.[8, 19] **FCC2** and **FCC3**, reconstructions were obtained with 1200 iterations of the difference map algorithm[9] followed by 100 iterations of maximum likelihood.[8, 19] Reconstructions were performed by sharing information on the illumination between consecutive scans taken at different projection angles.[14, 20] Prior to tomography reconstructions, the complex-valued projections were aligned and processed as described in Guizar-Sicairos *et al.*[21]. Horizontal alignment was ensured based on tomographic consistency.[22] Tomographic reconstruction of phase projections was performed using a modified filtered back-projection algorithm (FBP).[21] To mitigate noise in the reconstruction, a Hanning filter was used. The obtained tomograms provide the 3D distribution of the refractive index decrement, $\delta(\mathbf{r})$, which away from absorption edges, yields directly the electron density.[7, 10]

Estimation of Spatial Resolution

The half period spatial resolution of ptychographic tomograms was estimated by Fourier shell correlation (FSC).[1] The full dataset of angular projections used for the tomographic reconstructions was divided in half, and two independent tomograms with double angular spacing were reconstructed independently. Then, the correlation between these two tomograms in Fourier domain was calculated and the resolution estimated based on the intersection with a set threshold. The threshold criteria for the FSC was the ½ bit criterion.[1] FSC line plots of FCC1, FCC2 and FCC3 tomograms are shown in Supplementary Figure 2. The spatial resolution of the FCC1 tomogram is estimated to be 31 nm. The spatial resolution of FCC2 is estimated to be 44 nm. The spatial resolution of FCC3 is estimated to be 35 nm.

Analysis of Ptychographic Tomograms

Image segmentation, analysis, and all 3D renderings were carried out using the commercial **FEI Avizo® Fire**.

Matching of Material Phases: Component matching was achieved in part by comparing calculated electron densities of known catalyst components with measured electron density histograms of isolated components. Electron densities were calculated as described by Diaz *et al.*[10] As a result of correlated noise introduced by ptychographic reconstructions, the ptychographic tomograms possess an uncertainty in the determination of the electron density. A comparison of the retrieved electron density values of air with the tabulated density of air reveals a spread of 5-10% on single-pixel level.

The theoretical electron densities of hydrocarbon deposits (C_xH_y) were approximated with a density of 1.2 g cm^{-3} and a molecular weight of 18 g mol^{-1}. Those of Clay and ASA — being an inter-dispersed amorphous mixture of silica and alumina with unknown composition

— were approximated using a mass density of 3.1 g cm^{-3}, molecular weight of 80 g mol^{-1} and atomic number of 29. Because of its microporous morphology, the retrieved electron density of zeolites is expectedly lower than the calculated electron density. This is a result of partial-volume effects, where a mixture of material components occupies a single voxel.

To estimate the precision of the tomogram-provided electron densities we first isolated visually pure elements of air, pores, zeolite and clay/ASA that are roughly equal in volume, ~(250-300 $nm)^3$, extracted the corresponding histograms and calculated the respective mean, $\tilde{x}$, standard deviation, σ, and the confidence interval, CI, at the 95% confidence level of these elements. For air these are 0.0002 $Å^{-3}$ $\tilde{x}$| 0.036 σ| 0.0013 CI. The increased spread for pores (0.0209 $Å^{-3}$ $\tilde{x}$| 0.071 σ| 0.0022 CI), zeolite (0.454 $Å^{-3}$ $\tilde{x}$| 0.081 σ| 0.0030 CI) and clay/ASA (0.660 $Å^{-3}$ $\tilde{x}$| 0.055 σ| 0.0018 CI) are attributed to additional component inhomogeneity and partial-volume effects. Data was taken from tomogram FCC3.

Isolation of FCC Particles: The air surrounding the FCC particles in the acquired tomograms was removed by interactively thresholding the tomogram and morphological operations i.e. closing creating a binary mask.

Segmentation of Material Phases: Segmentation of ptychographic tomograms allowed the separation of pores, clay/ASA and zeolite components. This was followed by morphological operations to refine the segmentation.[23] From the intensity histogram shown in Figure 3a iv we chose thresholds placed midway between two adjacent peaks as starting thresholds.

To ascertain the correctness of selected thresholds in providing the most accurate material distribution possible, in particular with regards to zeolite and clay/ASA bearing the highest risk of miss-classifying components, we shifted the corresponding segmentation threshold by half a zeolite standard deviation either towards zeolite or clay. The zeolite standard deviation was chosen as it showed the highest spread. Evident was that the general sample characteristics that influence the main conclusions predominantly remain present. In particular, the ASA shell is detected throughout, regardless of segmentation threshold. However, a comparison of the segmented tomograms with the provided electron density tomograms clearly showed that a divergence from the selected segmentation threshold leads to an increasing misidentification of zeolite or clay, or an overrepresentation of these phases, respectively.

Inter-particle distances of clay/ASA and zeolite and distances to the particle exterior were calculated using Euclidean distance maps.

Pore Size Distributions and Pore Analysis: Neighborhood-based component labelling was used to identify continuous pore regions. The dominant pore network in each composite was then subjected to a thickness analysis.[24] This method transforms the segmented image into a map where the intensity in each voxel in the porosity regions is replaced by the diameter of the largest sphere, which can entirely fit into the porous region surrounding the voxel. In such a way, a pore diameter map is created, which can be used to obtain the distribution of pore sizes independent of any additional model constraints.[25] The smallest spheres considered in the calculation of pore size distributions were 31 nm in diameter. This was done to account for the finite tomogram resolution and to minimize mean thickness effects in the normalized pore size distributions as outlined in Hildebrand and Rüegsegger [24].

Propagation Distance and Characteristic Diffusion Length: The maximum propagation distance within the pore network of FCC1 was calculated using Avizo Fire. Once the pore network was segmented, permeable and impermeable areas in the tomogram were defined accordingly. We then cropped the tomogram with respect to the diffusion highway entrance furthest from the particle center. This entrance was then set as the starting point of a unidirectional flood fill operation with permeation limited to the pore network. The obtained distance map represents the maximum distance between particle exterior and active site.

The characteristic 1D diffusion length (L) of a feed molecule was calculated according to $\tau = L^2/\kappa$, where κ is the characteristic time scale and τ is the diffusion coefficient.[26]

Supplementary References

1. van Heel M., Schatz M. Fourier shell correlation threshold criteria. *Journal of Structural Biology* **151**, 250-262 (2005).

2. Diamond S. Mercury porosimetry: An inappropriate method for the measurement of pore size distributions in cement-based materials. *Cement and Concrete Research* **30**, 1517-1525 (2000).

3. Wallenstein D., Fougret C., Brandt S., Hartmann U. Application of Inverse Gas Chromatography for Diffusion Measurements and Evaluation of Fluid Catalytic Cracking Catalysts. *Industrial & Engineering Chemistry Research* **55**, 5526-5535 (2016).

4. Gualtieri A., Bellotto M., Artioli G., Clark S.M. Kinetic study of the kaolinite-mullite reaction sequence. Part II: Mullite formation. *Physics and Chemistry of Minerals* **22**, 215-222 (1995).

5. Yaluris G., Cheng W.C., Peters M., McDowell L.T., Hunt L. Mechanism of fluid cracking catalysts deactivation by Fe. *Studies in Surface Science and Catalysis* **149**, 139-163 (2004).

6. Gualtieri A.F., Moen A., Nicholson D.G. XANES study of the local environment of iron in natural kaolinites. *European Journal of Mineralogy* **12**, 17-23 (2000).

7. Dierolf M., Menzel A., Thibault P., Schneider P., Kewish C.M., Wepf R., Bunk O., Pfeiffer F. Ptychographic X-ray computed tomography at the nanoscale. *Nature* **467**, 436-439 (2010).

8. Guizar-Sicairos M., Fienup J.R. Phase retrieval with transverse translation diversity: a nonlinear optimization approach. *Optics Express* **16**, 7264-7278 (2008).

9. Thibault P., Dierolf M., Menzel A., Bunk O., David C., Pfeiffer F. High-Resolution Scanning X-ray Diffraction Microscopy. *Science* **321**, 379-382 (2008).

10. Diaz A., Trtik P., Guizar-Sicairos M., Menzel A., Thibault P., Bunk O. Quantitative x-ray phase nanotomography. *Physical Review B* **85**, 020104 (2012).

11. da Silva J.C., Trtik P., Diaz A., Holler M., Guizar-Sicairos M., Raabe J., Bunk O., Menzel A. Mass Density and Water Content of Saturated Never-Dried Calcium Silicate Hydrates. *Langmuir* **31**, 3779-3783 (2015).

12. Gorelick S., Vila-Comamala J., Guzenko V.A., Barrett R., Salomé M., David C. High-efficiency Fresnel zone plates for hard X-rays by 100 keV e-beam lithography and electroplating. *Journal of Synchrotron Radiation* **18**, 442-446 (2011).

13. Kraft P., Bergamaschi A., Bronnimann C., Dinapoli R., Eikenberry E.F., Graafsma H., Henrich B., Johnson I., Kobas M., Mozzanica A., Schleputz C.M., Schmitt B. Characterization and Calibration of PILATUS Detectors. *IEEE Transactions on Nuclear Science* **56**, 758-764 (2009).

14. Holler M., Diaz A., Guizar-Sicairos M., Karvinen P., Färm E., Härkönen E., Ritala M., Menzel A., Raabe J., Bunk O. X-ray ptychographic computed tomography at 16 nm isotropic 3D resolution. *Scientific Reports* **4**, 3857 (2014).

15. Holler M., Raabe J. Error motion compensating tracking interferometer for the position measurement of objects with rotational degree of freedom. *Optical Engineering* **54**, 054101-054101 (2015).

16. Huang X., Yan H., Harder R., Hwu Y., Robinson I.K., Chu Y.S. Optimization of overlap uniformness for ptychography. *Optics Express* **22**, 12634-12644 (2014).

17. Kaestner A., Münch B., Trtik P., Butler L. Spatiotemporal computed tomography of dynamic processes. *Optical Engineering* **50**, 123201-123209 (2011).

18. Howells M.R., Beetz T., Chapman H.N., Cui C., Holton J.M., Jacobsen C.J., Kirz J., Lima E., Marchesini S., Miao H., Sayre D., Shapiro D.A., Spence J.C.H., Starodub D. An assessment of the resolution limitation due to radiation-damage in X-ray diffraction microscopy. *Journal of Electron Spectroscopy and Related Phenomena* **170**, 4-12 (2009).

19. Thibault P., Guizar-Sicairos M. Maximum-likelihood refinement for coherent diffractive imaging. *New Journal of Physics* **14**, 063004 (2012).

20. Guizar-Sicairos M., Johnson I., Diaz A., Holler M., Karvinen P., Stadler H.-C., Dinapoli R., Bunk O., Menzel A. High-throughput ptychography using Eiger: scanning X-ray nano-imaging of extended regions. *Optics Express* **22**, 14859-14870 (2014).

21. Guizar-Sicairos M., Diaz A., Holler M., Lucas M.S., Menzel A., Wepf R.A., Bunk O. Phase tomography from x-ray coherent diffractive imaging projections. *Optics Express* **19**, 21345-21357 (2011).

22. Guizar-Sicairos M., Boon J.J., Mader K., Diaz A., Menzel A., Bunk O. Quantitative interior x-ray nanotomography by a hybrid imaging technique. *Optica* **2**, 259-266 (2015).

23. Gonzalez R.C., Woods R.E. *Digital Image Processing (3rd Edition)*. Prentice-Hall, Inc. (2006).

24. Hildebrand T., Rüegsegger P. A new method for the model-independent assessment of thickness in three-dimensional images. *Journal of Microscopy* **185**, 67-75 (1997).

25. da Silva J.C., Mader K., Holler M., Haberthür D., Diaz A., Guizar-Sicairos M., Cheng W.-C., Shu Y., Raabe J., Menzel A., van Bokhoven J.A. Assessment of the 3 D Pore Structure and Individual Components of Preshaped Catalyst Bodies by X-Ray Imaging. *ChemCatChem* **7**, 413-416 (2015).

26. Kirby B.J. *Micro- and Nanoscale Fluid Mechanics*. Cambridge University Press (2010).
