# Supplementary material for: A three-dimensional view of structural changes caused by deactivation of fluid catalytic cracking catalysts
Source: Nat Commun. 2017 Oct 9;8:809. doi: 10.1038/s41467-017-00789-w (PMC5634498; doi:10.1038/s41467-017-00789-w)
Supplement: Supplementary file 3 — Description of Additional Supplementary Files [file 41467_2017_789_MOESM3_ESM.pdf]

Description of Additional Supplementary Files

File Name: Supplementary Movie 1
Description: Volume Rendering of Ptychographic Tomogram FCC1. Shown are volume reconstruction of and cut slices through the retrieved electron density map, the component segmented tomogram, calculated thickness map and identifiable diffusion highways in FCC1. The beam direction is along the z axis, with the y axis vertical, while the sample holder is placed normal to the beam direction (z).

File Name: Supplementary Movie 2
Description: Volume Rendering of Ptychographic Tomogram FCC2. Shown are volume reconstruction of and cut slices through the retrieved electron density map, the component segmented tomogram, calculated thickness map and identifiable diffusion highways in FCC2. The beam direction is along the z axis, with the y axis vertical, while the sample holder is placed normal to the beam direction (z).

File Name: Supplementary Movie 3
Description: Volume Rendering of Ptychographic Tomogram FCC3. Shown are volume reconstruction of and cut slices through the retrieved electron density map, the component segmented tomogram, calculated thickness map and identifiable diffusion highways in FCC3. The beam direction is along the z axis, with the y axis vertical, while the sample holder is placed normal to the beam direction (z).
